# Simultaneous Identification of DNA and RNA Viruses Present in Pig Faeces Using Process-Controlled Deep Sequencing

**DOI:** 10.1371/journal.pone.0034631

**Published:** 2012-04-13

**Authors:** Jana Sachsenröder, Sven Twardziok, Jens A. Hammerl, Pawel Janczyk, Paul Wrede, Stefan Hertwig, Reimar Johne

**Affiliations:** 1 Federal Institute for Risk Assessment, Berlin, Germany; 2 Institute for Molecular Biology and Bioinformatic, Charite, Berlin, Germany; Institute for Animal Health, United Kingdom

## Abstract

**Background:**

Animal faeces comprise a community of many different microorganisms including bacteria and viruses. Only scarce information is available about the diversity of viruses present in the faeces of pigs. Here we describe a protocol, which was optimized for the purification of the total fraction of viral particles from pig faeces. The genomes of the purified DNA and RNA viruses were simultaneously amplified by PCR and subjected to deep sequencing followed by bioinformatic analyses. The efficiency of the method was monitored using a process control consisting of three bacteriophages (T4, M13 and MS2) with different morphology and genome types. Defined amounts of the bacteriophages were added to the sample and their abundance was assessed by quantitative PCR during the preparation procedure.

**Results:**

The procedure was applied to a pooled faecal sample of five pigs. From this sample, 69,613 sequence reads were generated. All of the added bacteriophages were identified by sequence analysis of the reads. In total, 7.7% of the reads showed significant sequence identities with published viral sequences. They mainly originated from bacteriophages (73.9%) and mammalian viruses (23.9%); 0.8% of the sequences showed identities to plant viruses. The most abundant detected porcine viruses were kobuvirus, rotavirus C, astrovirus, enterovirus B, sapovirus and picobirnavirus. In addition, sequences with identities to the chimpanzee stool-associated circular ssDNA virus were identified. Whole genome analysis indicates that this virus, tentatively designated as pig stool-associated circular ssDNA virus (PigSCV), represents a novel pig virus.

**Conclusion:**

The established protocol enables the simultaneous detection of DNA and RNA viruses in pig faeces including the identification of so far unknown viruses. It may be applied in studies investigating aetiology, epidemiology and ecology of diseases. The implemented process control serves as quality control, ensures comparability of the method and may be used for further method optimization.

## Introduction

About 191 million pigs are kept in Europe as farm animals [1]. As they provide a crucial source of food worldwide, viruses directly affecting the pig health have a great impact on food production in general. In addition, pigs are a natural habitat for zoonotic viruses, which can cause infectious diseases in humans, such as influenza virus [2], rotavirus [3] and hepatitis E virus [4]. Other viruses present in pigs such as bacteriophages or viruses causing subclinical infections may affect the pig health indirectly, e.g. by modification of the bacterial population or by modulation of the immune system as also shown for other hosts [5], [6]. In order to understand the complex interaction between the different viruses, their host cells and the immune system, a simultaneous analysis of a broad range of virus species in a distinct compartment is desirable. For a long time, such an analysis was hampered by technical limitations [7]. Recently, this problem was solved by the development of novel molecular approaches utilizing deep sequencing techniques, e.g. 454 pyrosequencing [8]. This technique enables the simultaneous analysis of thousands of sequences present in a DNA sample. Deep sequencing techniques have been frequently used to study the composition of microbial communities in different kinds of environmental samples including faeces of humans and animals [9]–[11]. They have also been applied to the detection of single known and unknown viruses in various kinds of samples [12]–[16].

Some studies have applied deep sequencing methods to the analysis of the composition of viral communities in human faeces [17]–[21], but only one study focussed on pigs [22]. In most cases, the majority of detected viruses were bacteriophages. Among the RNA viruses, transitory plant viruses, which most probably originated from feed, were often identified. In addition, several human/animal viruses were detected.

The described protocols used for the analysis of the composition of the intestinal viral community generally consist of four basic steps: (i) purification and concentration of the virus particles present in the faeces, (ii) extraction of nucleic acids, (iii) deep sequencing of the nucleic acid and (iv) bioinformatic analysis of the sequence data. Despite this common backbone, the protocols differ from each other in several details. For example, different methods are used for purification and concentration of virus particles. As the virus particles are very heterogeneous in shape and size [23], the applied filtration method will strongly influence the result of the analysis. Moreover, the genomes of viruses consist of either DNA or RNA, which additionally may appear in different topologies [23]. Most of the protocols analyse DNA and RNA separately [13], [17], [21], [22], which makes the comparison between both groups of viruses difficult. In many of the protocols, various amplification steps, which may affect the distribution of the different genome types, are implemented. In addition, either primary sequence reads or contigs assembled from these sequence reads were used for analysis. All of these variations in the applied protocols may lead to different results. Indeed, the reported composition of the human viral gut community varies remarkably, which - beside other factors - may also be caused by application of different protocols for its analysis [17]–[19], [21].

Here, a protocol was established for the simultaneous analysis of DNA and RNA viruses present in pig faeces. A process control consisting of a mixture of bacteriophages with different morphology and genome type was added to the sample and used to assess the efficiency of the method. The use of a process control may enable the optimization of the method as well as a comparison to other published protocols. The optimized protocol was thereafter tested with a pooled pig faecal sample in order to analyse the composition of the viruses present in pig faeces.

## Results

### Establishment of a process control

To monitor the performance of the method and to optimize the distinct steps of the purification/concentration method, we used three bacteriophages (T4, M13, MS2) with different morphology and genome type [23] as process control (Tab. 1). Bacteriophages were chosen as they are easy to propagate and do not need extensive safety containment. T4 belongs to the family *Myoviridae* and has a large, double-stranded DNA. M13 is a filamentous phage and contains a small genome of single-stranded DNA. MS2 has a small icosahedral capsid containing a genome of single-stranded RNA. Using these three bacteriophages, the most common genome types were covered by the process control. All phages were added to the pig faecal suspension at a defined concentration. Quantitative real-time RT-PCR (qRT-PCR) protocols were established for these bacteriophages (Tab. 2), which enabled detection and quantification of the viruses at each step of purification. By comparison of the determined genome copy number at the end of the purification process with the number at the beginning, the efficacy of the purification protocol was calculated.

### Optimization of the protocol for the detection of the viral community in pig faeces

**Figure 1:**
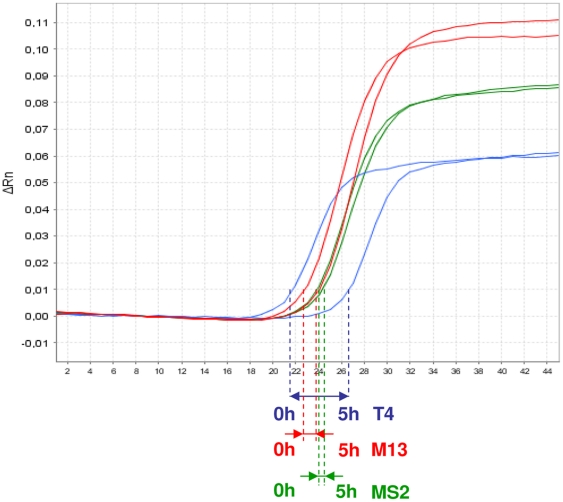
Optimization of the purification protocol using qRT-PCR detection of bacteriophages used as process control. The amplification curves determined by qRT-PCR for the phages T4 (blue), M13 (red) and MS2 (green) were determined before and after centrifugation for 17,000× g for 5 hours. The shift between the curves, which corresponds to the degree of virus loss, is indicated by arrows coloured according to the bacteriophage type.

Using the established process control, the method for purification of virus particles from pig faeces was gradually optimized. Several techniques were tested and various conditions were compared to each other, e.g. magnetic stirring vs. stomaching of the faecal sample, filtration vs. centrifugation for removal of larger debris, classical filtration vs. tangential flow filtration (TFF) for removal of smaller debris and bacteria, TFF vs. ultrafiltration for concentration of virus particles and caesium chloride gradient ultracentrifugation vs. sucrose gradient centrifugation for final purification and concentration of virus particles (data not shown). For example, a prolonged centrifugation time for removal of larger debris resulted in a marked loss of bacteriophage T4, whereas bacteriophages M13 and MS2 were less affected. Figure 1 shows the original qRT-PCR plots of the three bacteriophages from this purification step before and after centrifugation at 17,000× g for 5 hours. However, further processing of the sample by TFF without prior centrifugation was not practicable because of the appearance of filter occlusions. Therefore, a shorter centrifugation of three hours was used, although this might result in some loss of virus particles with high densities.

**Table 1:** Properties of the bacteriophages T4, M13 and MS2 used as process control.

	T4	M13	MS2
**family**	*Myoviridae*	*Inoviridae*	*Leviviridae*
**particle shape**	Icosahedral head+ contractile tail	filamentous	icosahedral
**particle size**	Ø 100 nm+ 100 nm tail	1000 nm long	Ø 30 nm
**genome** 1	dsDNA	ssDNA	ssRNA
**genome size[kb]**	169	6.5 (circular)	3.6
**density in CsCl [g/ml]**	1.5	1.4	1.4
**reads**	29	137	175
**contigs**	/	4	5
**average contig size**		647	694

1 ssDNA – single-stranded DNA; dsDNA – double-stranded DNA; ssRNA – single-stranded RNA

**Table 2:** Primers and probes used for qPCR detection of the three bacteriophage genomes.

	sequence (5′–3′)	reference
	probe: FAM-TTG GGC GCG GTA ATG ATT CCT ACG-TAMRA	
**M13**	for: ACG CCT CGC GTT CTT AGA ATA CC	this study
	rev: ACC GCA CTC ATC GAG AAC AAG C	
	probe: FAM-CCT TTT TAG CTG CTT TAG TTT CTG C-TAMRA	
**T4**	for: GTA TCA GCA TCT TTA CCG CA	this study
	rev: GCT TTG GCT CGT AAA TTG GC	
	probe: FAM-ACC TCG GGT TTC CTG CTT GCT CGT-TAMRA	
**MS2**	for: GGC TGC TCG CGG ATA CCC	[28]
	rev: TGA GGG AAT GTG GGA ACC	

After the purification step had been optimized, several parameters were tested for efficient removal of free nucleic acids from the purified virus particle suspension. While DNase I digestion had only little effects on the control bacteriophages, treatment with RNase A led to a significant decrease in the detectable genome number of bacteriophage MS2, which may be explained by the presence of residual RNase activity in the final nucleic acid preparation. Therefore, treatment with RNase was omitted in the final protocol. Tests with naked RNA prepared from bacteriophage MS2 that was added to pig faecal samples showed that the RNA was no longer detectable by qRT-PCR after 30 minutes of incubation at room temperature, suggesting that RNase treatment is not necessary at later steps.

**Figure 2:**
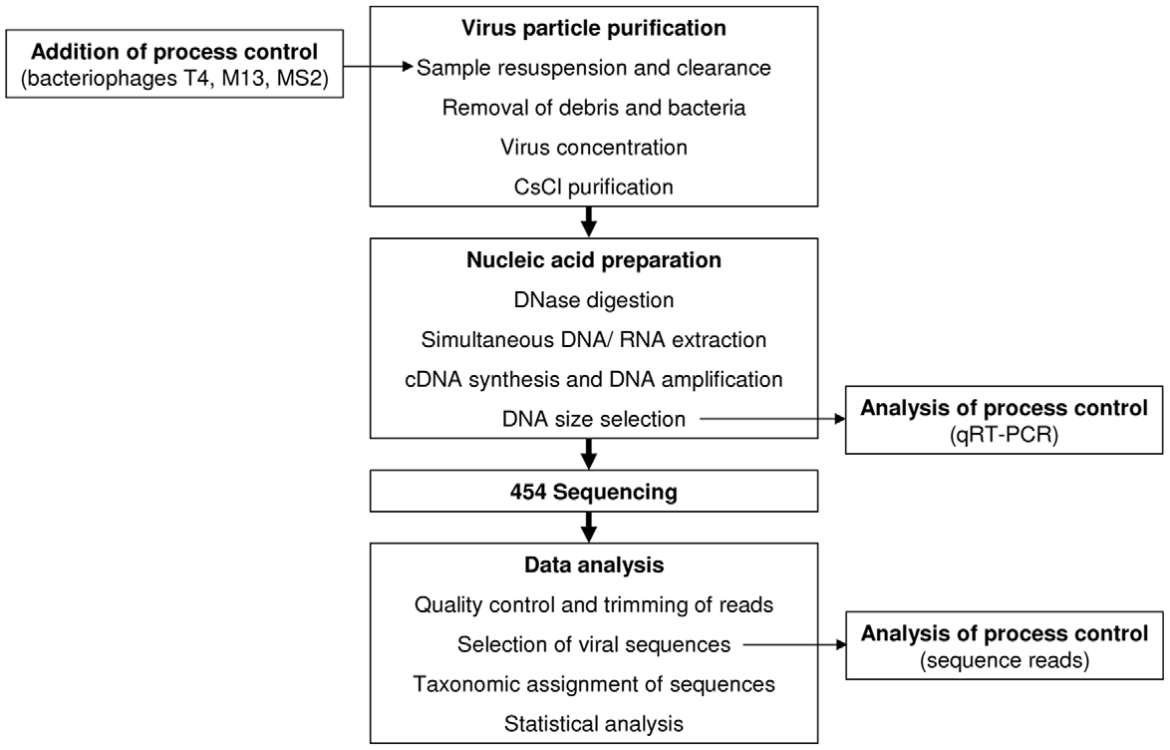
Schematic presentation of the general steps for analysis of the total fraction of virus particles from pig faeces by deep sequencing. The addition of the process control (bacteriophages T4, M13, MS2) and the subsequent points of analysis are indicated.

Although nucleic acid amplification should be omitted to avoid errors caused by PCR, the optimized method contains an amplification step at the end of the procedure. This step had to be introduced, because the yield of nucleic acids was too low for direct use in deep sequencing. In order to minimize the amplification steps, aliquots were taken from different PCR cycles to determine the minimum amplification needed. A general scheme of the working steps in the optimized protocol is presented in Fig. 2. Applying this protocol, recovery rates of 4%, 125% and 105% were obtained for the genomes of T4, M13 and MS2, respectively, by comparing qRT-PCR results after addition of the phages with those from the final nucleic acid preparation. Recovery rates higher than 100% can be explained by concentration of the virus particles and by amplification of their genomes during the last step of the protocol. The low recovery rate of T4 may be explained by loss of this high density virus during clearing centrifugation as shown above.

### Application of the optimized method to a pooled pig faecal sample

The faeces of five 35 day-old male pigs were pooled and subjected to purification of virus particles using the optimized protocol. By deep sequencing of the extracted nucleic acid, 69,613 reads with an average sequence length of 250 nucleotides were generated. After trimming of the sequence ends and exclusion of reads shorter than 50 nucleotides, 66,129 reads were included in the analyses. From these reads, 1,482 contigs could be assembled with a length between 100 bp and 1998 bp. The primary sequencing data are summarized in Table 3.

**Table 3:** Primary data obtained for the pig faecal sample after deep sequencing.

**total number of reads**	69,613
**number of reads included in analysis**	66,129
**average read length (bp)**	250
**total number of bases (Mb)**	17,5
**number of contigs**	1,482
**contigs >500 bp**	321

As a quality control of the method, the sequence reads were analysed for the presence of nucleotide sequences originating from the added bacteriophages T4, M13 and MS2. Sequences of all three phages could be identified. In detail, 175 reads originated from MS2, 137 reads from M13 and 29 reads from T4 (Tab. 1), thus correlating with the numbers of the bacteriophage genomes detected by qRT-PCR in the final preparation. These reads were excluded from the following data analysis.

### Data analysis

Primary reads and contigs were checked for sequence identities with a viral genome database including the calculation of tBLASTx E scores. Only sequences with a tBLASTx score E < = 10^−4^ were suspected to represent sequences of known and closely related viruses. By this, 5,366 primary reads (7.7%) and 315 contigs (21.3%) showed significant identities to known viral sequences and were therefore included in the further data analysis.

**Figure 3:**
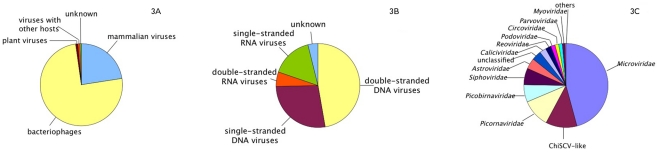
Summary of results for the detection of viruses in a pooled faecal sample derived from five 35 day-old male pigs. The numbers of generated viral sequences are analysed according to virus host (A), viral genome type (B) or assignment to a specific virus family (C). In the latter case, the numbers have been corrected according to the genome length of the respective virus species (see Text for details).

Out of the selected 5,366 primary reads, 3,965 reads (73.9%) showed significant sequence identities to bacteriophages, 1,282 reads (23.9%) were similar to mammalian viruses, 43 reads (0.8%) were identified as plant virus sequences and 56 reads (1.4%) could not be classified into one of these groups (Fig. 3A). An assignment of the primary reads to different types of viral genomes identified 49.8% double-stranded (ds) DNA viruses, 26.4% single-stranded (ss) DNA viruses, 6.0% dsRNA viruses, 13.5% ssRNA viruses and 4.2% unknown genome types (Tab. 4, Fig. 3B). By analysis of the contigs, 39.0% dsDNA viruses, 36.2% ssDNA viruses, 6.3% dsRNA viruses, 14.3% ssRNA viruses and 4.1% unknown genome types were detected. An assignment of the sequences to known viral families was performed by application three different calculations (Tab. 5). Using the primary reads only, the most abundant virus families were *Siphoviridae* (30.1%), *Microviridae* (21.7 %) and *Myoviridae* (11.3%), all of them representing bacteriophages. Using the contigs only, three bacteriophage families were again most prominent, but with different percentages: 33.1% *Siphoviridae*, 14.9% *Myoviridae* and 9.1% *Podoviridae*. The maximum identities between the deduced amino acid sequences of the contigs with the lowest tBLASTx E scores and that of known members of the respective virus families ranged from 35% to 100% (Tab. 5). The lengths of the viral genomes are remarkably different between different virus families [23] and it can be assumed that longer genomes are represented by more primary reads per genome than smaller ones. Therefore, a correction factor for the genome length was introduced to give estimation for the particle number. After this correction, the most abundant viral families were *Microviridae* (bacteriophages, 45.9%) followed by the Chimpanzee stool associated circular ssDNA virus (ChiSCV, see below, 12.0%) and *Picornaviridae* (mammalian viruses, 10.8%) (Fig. 3C). The Shannon index was used to assess the diversity of the sequences detected in the sample. Shannon indexes of 4.7777 and 4.252 were calculated for the primary reads and contigs, respectively.

**Table 4:** Numbers of viral sequences detected in the pig faecal sample, according to genome types.

	Reads		contigs	
genome type^1^	number of reads (% of reads)	number of different virus species	number of contigs (% of contigs)	number of different virus species
dsDNA	2674 (49.8)	379	123 (39.0)	85
ssDNA	1419 (26.4)	29	114 (36.2)	20
dsRNA	323 (6.0)	21	20 (6.3)	3
ssRNA	726 (13.5)	17	45 (14.3)	9
unknown	224 (4.2)	20	13 (4.1)	5
Total	5366 (100.0)	466	315 (100.0)	121

1 dsDNA – double-stranded DNA; ssDNA – single-stranded DNA; dsRNA – double-stranded RNA; ssRNA – single-stranded RNA

**Table 5:** Number of viral sequences detected in the pig faecal sample, according to virus family.

contigs			Reads		reads/genome size	
	% of contigs	sequence identity* (length of compared sequence)		% of reads		% families (corrected)
*Siphoviridae*	33.06	75% (99aa)	*Siphoviridae*	30.06	*Microviridae*	45.86
*Myoviridae*	14.88	62% (102aa)	*Microviridae*	21.71	ChiSCV-like	12.01
*Podoviridae*	9.09	54% (125 aa)	*Myoviridae*	11.33	*Picornaviridae*	10.76
unclassified**	9.09	45% (80aa)	*Picornaviridae*	8.83	*Picobirnaviridae*	6.59
*Microviridae*	6.61	54% (206 aa)	unclassified**	6.78	*Siphoviridae*	6.38
*Astroviridae*	3.31	52% (182 aa)	*Podoviridae*	4.71	*Astroviridae*	4.17
*Circoviridae*	3.31	45% (58 aa)	*Reoviridae*	4.38	unclassified**	3.71
*Parvoviridae*	3.31	55% (77aa)	ChiSCV-like	3.24	*Caliciviridae*	2.39
*Phycodnaviridae*	2.48	42% (45aa)	*Astroviridae*	2.83	*Reoviridae*	2.28
*Picornaviridae*	1.65	87% (200aa)	*Caliciviridae*	1.83	*Podoviridae*	1.77
*Reoviridae*	1.65	93% (271aa)	*Picobirnaviridae*	1.62	*Circoviridae*	1.33
*Herpesviridae*	1.65	45% (29aa)	*Parvoviridae*	0.65	*Parvoviridae*	1.21
*Poxviridae*	1.65	100% (42aa)	*Phycodnaviridae*	0.58	*Myoviridae*	1.20
ChiSCV-like	0.83	68% (105aa)	*Poxviridae*	0.52	*Inoviridae*	0.07
*Caliciviridae*	0.83	92% (99aa)	*Circoviridae*	0.26	*Tectiviridae*	0.06
*Picobirnaviridae*	0.83	42% (86aa)	*Mimiviridae*	0.24	*Bromoviridae*	0.05
*Mimiviridae*	0.83	48% (42aa)	*Herpesviridae*	0.11	*Luteoviridae*	0.03
*Potyviridae*	0.83	50% (28aa)	*Tectiviridae*	0.09	*Phycodnaviridae*	0.02
*Phaeovirus*	0.83	42% (57aa)	*Iridoviridae*	0.09	*Poxviridae*	0.02
*Inoviridae*	0.83	29% (52aa)	*Inoviridae*	0.06	*Herpesviridae*	0.006
*Iridoviridae*	0.83	35% (78aa)	*Bromoviridae*	0.02	*Iridoviridae*	0.005
			*Luteoviridae*	0.02	*Bicaudaviridae*	0.002
			*Bicaudaviridae*	0.02	*Mimiviridae*	0.002

* sequence identity: Contigs were compared to known sequences of the indicated virus families and the respective contig with the lowest tBLASTx E score was used for calculation of deduced amino acid sequence identities.

** unclassified: The highest identities of these sequences were found to virus sequences, which have not been assigned to a virus family according to the data available in the GenBank database.

A closer inspection of the sequences revealed that a total of 15 different viruses with sequence identities to mammalian viruses were identified (Tab. 6). These included the RNA viruses: kobuvirus, rotavirus group A and C, astrovirus, enterovirus B, sapovirus, picobirnavirus, teschovirus, picornavirus, and the DNA viruses: circovirus, bocavirus, pox virus, parvovirus and herpesvirus. In addition to these virus families, which had already been detected in pigs previously, a novel virus showing sequence identity to ChiSCV was detected and analysed in detail.

**Table 6:** Data on mammalian viruses detected in the pig faecal sample.

		Reads		contigs		
virus	genome type	number of reads (% of reads)	% of mammalian viruses	number of contigs (% of contigs)	% of mammalian viruses	sequence identity* (length of compared sequence)
Kobuvirus	ssRNA	330 (5.8)	25.7	19 (15.4)	21.6	87% (200aa)
Rotavirus C	dsRNA	207 (3.6)	16.1	16 (13.0)	18.2	93% (271aa)
ChiSCV	ssDNA	174 (3.0)	13.6	11 (8.9)	12.5	68% (271aa)
Astrovirus	ssRNA	152 (2.7)	11.9	13 (10.6)	14.8	52% (182aa)
Enterovirus	ssRNA	134 (2.3)	10.5	9 (7.3)	10.2	94% (135aa)
Sapovirus	ssRNA	98 (1.7)	7.6	3 (2.4)	3.4	87% (158aa)
Picobirnavirus	dsRNA	87 (1.5)	6.8	3 (2.4)	3.4	49% (49aa)
Rotavirus A	dsRNA	28 (0.5)	2.2	1 (0.8)	1.1	79% (76aa)
Bocavirus	ssDNA	27 (0.5)	2.1	3 (2.4)	3.4	61% (46aa)
Poxvirus	dsDNA	15 (0.3)	1.2	2 (1.6)	2.3	100% (42aa)
Parvovirus	ssDNA	8 (0.1)	0.6	1 (0.8)	1.1	55% (77aa)
Teschovirus	ssRNA	7 (0.1)	0.5	/	/	/
Circovirus	ssDNA	7 (0.1)	0.5	5 (4.1)	5.7	41% (85aa)
Herpesvirus	dsDNA	5 (0.1)	0.4	2 (1.6)	2.3	22% (95aa)
Picornavirus	ssRNA	3 (0.1)	0.2	/	/	/
total		1282 (22.5)	100.0	88 (7.5)	100.0	/

* Contigs were compared to known sequences of the indicated virus families and the respective contig with the lowest tBLASTx E score was used for calculation of deduced amino acid sequence identities.

### Whole genome analysis of a novel circular ssDNA virus

**Figure 4:**
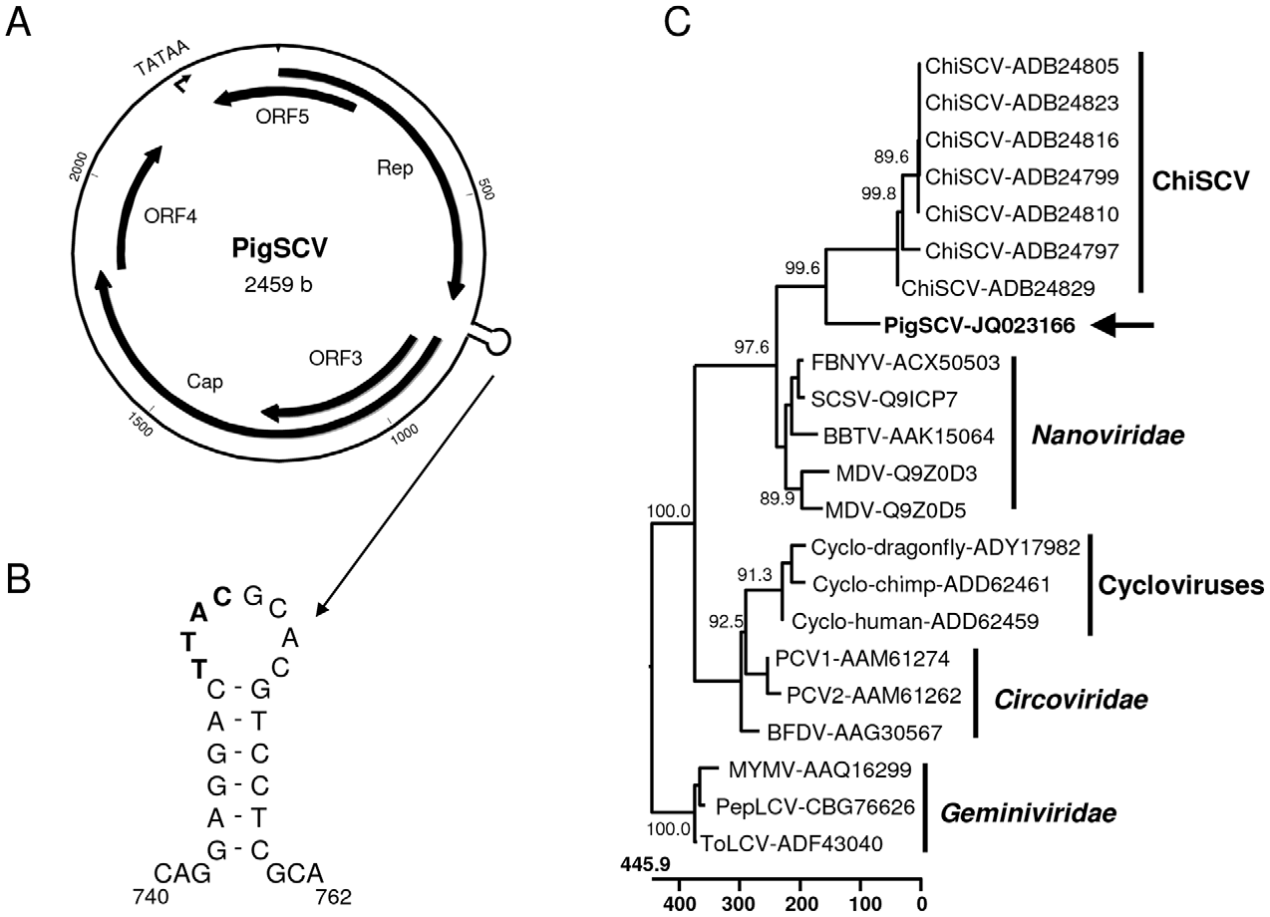
Genome analysis of the novel pig stool-associated single-stranded DNA virus (PigSCV) detected in the faecal sample. (A) Genome map: ORFs encoding the replication-associated protein (Rep), the capsid protein (Cap) and several unknown proteins (ORF3-5), the position of a putative TATA-box and the stemloop structure are indicated. (B) Proposed secondary structure of the stem-loop structure; nucleotides conserved in PigSCV and ChiSCV are shown in bold face. (C) Phylogenetic tree constructed on the basis of the deduced amino acid sequences of the Rep protein of selected circular ssDNA viruses of animals and plants. The position of PigSCV is indicated by an arrow.

The whole genome of the suspected ChiSCV-related virus was amplified from the nucleic acid preparation in two fragments using PCR. Primers used in PCR were designed on the basis of the sequences derived from the deep sequencing analysis. After sequencing of the overlapping PCR products, the whole genome sequence was assembled and ORFs were identified. The genome of this virus has a length of 2,459 nucleotides and a circular topology (Fig. 4A). A total of 6 ORFs encoding proteins consisting of more than 100 amino acids were identified. The protein encoded by ORF1 shows the highest amino acid sequence identity (27.0%) to the replicase protein (Rep) of ChiSCV (Gen-Bank Acc.-No. ABD24829.1). ORF2 shows the highest amino acid sequence identity (49.2%) to the capsid protein (Cap) of ChiSCV (Gen-Bank Acc.-No. ADB24798.1). No significant similarities to known proteins could be determined when the amino acid sequences encoded by the other ORFs were analysed. The genome organization of PigSCV is different from that of ChiSCV: while Rep and Cap are encoded by the same DNA strand in ChiSCV, the respective genes are oriented in opposite direction in the PigSCV genome (Fig. 4A). A search for repetitive sequences revealed a short stemloop sequence between nucleotide positions 743 and 759 on the PigSCV genome (Fig. 4B). This stemloop also contains a conserved sequence with identitiy to stemloop sequences of several ChiSCV isolates. A phylogenetic analysis of the amino acid sequence of Rep together with related viruses from animals and plants revealed the closest relationship of PigSCV to ChiSCV (Fig. 4C). However, within this group, the PigSCV sequence forms a separate branch indicating a membership of this virus to a separate species.

In order to analyze PigSCV infection in the individual pigs, stored faecal and serum samples from the animals were tested by PCR for the presence of the PigSCV genome. As evident from Table 7, specific PCR products were detected in faecal samples of each of the tested piglets; however, at different time-points and with different intensity (Tab. 7). The highest detection rate was found in the pigs at 33 and 35 days of age. The PigSCV genome was not detected in serum samples derived from the pigs at 42 days of age.

**Table 7:** Detection of the PigSCV genome in samples from pigs using PCR.

age (days)	29	33	35	42	
sample	faeces	Faeces	faeces	faeces	serum
piglet 1	−	+	−	+	−
piglet 2	−	n.d.	++	−	−
piglet 3	−	+	++	n.d.	−
piglet 4	−	++	++	−	−
piglet 5	++	−	+	−	−

− no band visible after electrophoresis of PCR product

+ faint band visible after electrophoresis of PCR product

++ strong band visible after electrophoresis of PCR product

n.d. – not done

## Discussion

The viral community present in animal faeces is complex in function and composition. So far, only little is known about the diversity and distribution of viruses in this ecosystem. The analysis of the composition of viruses in faeces was hampered for a long time by the lack of suitable methods. However, the recent development of deep sequencing techniques enabled the analysis of the viral community in faeces from different species like human [18], [19], [21], horse [24], turkey [25], sea lion [26] and rodents [27]. For pigs, only one study was published very recently [22]. The applied protocols are diverse and not standardized; therefore, comparison of the generated data is difficult.

To overcome the problem of comparability, we here suggest the use of a process control for monitoring the efficiency of the protocol applied for the analysis. The process control consists of a set of previously characterized viruses, of which a defined number is added to the sample and then followed during the process. Similar process controls are well established in other analytical applications, such as PCR analysis of food and environmental samples [28]–[30]. For the application in analysis of viral communities, the process control should reflect the variety of viruses with different morphologies and genome types, which can be suspected to be present in the sample. The three selected bacteriophages used here are diverse in these aspects and represent a large proportion of known viruses. However, some morphologies, e.g. enveloped spherical viruses, as well as some genome types, e.g. double-stranded RNA, are not present so far and may be added in future in order to improve the process control. As a proof of principle, we could demonstrate that all of the bacteriophages added to the sample could be identified in the sequences generated by deep sequencing. This indicates that the bacteriophages could be monitored throughout the whole process until its end, which includes sequence analysis. Using the recovery rates or sequence read numbers of the control bacteriophages, a comparison of results from studies performed with different techniques should be possible in the future. Although in principle the three used bacteriophages seem to be appropriate for monitoring the efficiency of the process, the low recovery rate of bacteriophage T4 demand further developments for a robust dsDNA virus control. Since the majority of viral sequences identified in our analysis of the pig faecal sample originate from dsDNA viruses, the bacteriophage T4 control may not sufficiently reflect the behaviour of many dsDNA viruses. Therefore, changes in the preparation protocol for bacteriophage T4 (e.g. by adding steps preventing aggregation of virus particles) or a replacement by another virus should be considered in the future.

The process control was also used as a read-out for optimization of the distinct steps of the method. By this, it could be confirmed that some of the techniques applied in former studies, e.g. tangential flow filtration [31], [32], are more effective than others. Besides minor variations of parameters in order to assess the optimal conditions for each step, some more basic decisions had to be made during the optimization process. One of them refers to the use of an RNase digestion step to remove contaminating free RNA from the virus particle suspension. As high amounts of RNase are difficult to inactivate or to remove from the preparation, residual RNase activity can destroy the viral RNA in the final nucleic acid preparation [32]. We show that free bacteriophage RNA is rapidly degraded in the faecal suspension within the first 30 minutes of the applied protocol; therefore, RNase treatment seems not necessary at later steps. A second decision refers to the use of amplification steps after preparation of the nucleic acids. It is known that some techniques induce changes in the relative distribution of viral genome types, e.g. several isothermal amplification methods preferentially amplify small circular DNA molecules [33]. To avoid extensive amplification steps, a relatively high amount of starting material (100 g faeces) was used here. However, the yield of purified virus nucleic acid was too low for direct use in deep sequencing. Therefore, amplification was implemented in the protocol; however, the cycles were limited to the lowest possible number. The identification of all known viral genome types in the primary sequence reads of the pig faecal sample analyzed here indicate that the optimized protocol is suitable for simultaneous identification of DNA viruses and RNA viruses in pig faeces.

By application of the optimized protocol to the pooled pig faecal sample, we detected 7.7% sequences with a high sequence identity to known virus genomes. This value is similar to that of recently published viral metagenome studies, which range from 2% [18] to 13% [22]. The slight difference between our study and that of Shan et al. [22], which both analyzed pig faecal samples, may be explained by the use of different databases and different sequence identity cut-offs. In other aspects, the results of both studies are similar. For example, Shan et al. identified 99% of the mammalian viruses as RNA viruses, which is similar to the value of 96% obtained in our study. Kobuvirus, enterovirus, sapovirus, teschovirus, astrovirus and bocavirus were identified in both studies. The similarity of these results is quite surprising taking into consideration that the samples analyzed in both studies originated from pigs from different continents. However, some differences still exist as coronavirus was only found by Shan et al. [22] and rotavirus was only detected in our study. The detection of rotaviruses, which are known as causative agents of gastroenteritis in pigs [34], [35], correlates with the fact that some of the pigs included in our study suffered from watery diarrhoea. In the study of Shan et al. [22], a correlation was found between the detection of bocavirus or coronavirus and the appearance of diarrhoea. Additionally, we detected a higher number of different virus families, compared to the study of Shan et al. [22], which may be explained by the use of a higher amount of faeces as starting material enabling detection of less represented viruses.

An additional factor with marked influence on the results of metagenomic analyses is the applied method for data analysis. First, a reliable cut-off for selection of sequences with significant sequence identities to known virus sequences has to be defined. Here, a tBLASTx E score < = 10^−4^ was used, according to other studies applying tBLASTx E scores between 10^−3^ [20], [21] and 10^−5^ [16], [18]. However, using this cut-off, many sequences with relative low deduced amino acid sequence identities were included in the analysis (Tab. 5). In addition, it has to be considered that the analysis of deduced amino acid sequences may not be appropriate in the case of sequences representing non-coding regions of viral genomes. Second, it should be decided, whether primary reads or contigs are used. In our study, the overall distribution of viral genome types or virus families was rather similar when using primary reads or contigs for analysis. However, analysis of the contigs only may lead to a reduction of detected virus diversity, as the rare sequences of less represented viruses cannot be assembled into contigs. Indeed, by using the primary reads we could identify 447 different virus species, compared to 121 viruses identified by the use of contigs. The Shannon index of diversity determined using the primary reads was 4.777 compared to 4.252 using contigs. As additionally contig assembly is strongly dependent on the used assembly algorithm [36], [37], it may be generally advisable to use primary reads for metagenomic data analysis. A third factor influencing results of data analysis refers to the highly diverse viral genome length. As a larger genome type is likely to be represented by more primary reads per genome than a smaller one, correction factors should be developed and included. We could show that the use of such correction factors can dramatically change the calculated distribution of viral families leading to a much higher abundance of mammalian viruses (with small genomes) than calculated without consideration of genome length.

By application of the optimized protocol and subsequent data analysis, sequences with similarities to a chimpanzee virus have been identified in the pig faeces. Subsequent amplification and analysis of the whole genome sequence indicate that this virus is closely related to the chimpanzee virus [38]. The two largest ORFs clearly show sequence similarities to the encoded replicase and capsid proteins of the chimpanzee virus, but their orientation in the genome is different and the percentages of the sequence identity are relatively low. As such virus sequences have not yet been described in pigs, it is concluded that it represents a novel pig virus. Analysis of samples from the pigs used in our experiment indicated that all of the piglets were infected with this virus, but the amounts and time-points of virus excretion in the faeces differed between the individuals. The different pattern of virus excretion in pigs receiving the same feed argues against an origin of the virus from infected plants that were ingested and excreted after intestinal passage, as originally suspected as one possibility for ChiSCV [38], although the feed could not be directly tested. No PigSCV genome was detected in the serum of the pigs; however, only one time-point could be analyzed because of the limited availability of the sera. Further studies are necessary in order to assess the origin, dissemination, organ distribution and clinical significance of this novel virus in pigs. PigSCV belongs to the single-stranded circular DNA viruses, which are increasingly detected in animals, plants and environmental samples [39]–[41]. The reason for this increased detection is unknown so far; however, it may be explained by the increased use of specific amplification methods in the detection protocols, which are known to amplify circular DNA molecules more efficiently than others [33]. Shan et al. [22] also found several single-stranded circular DNA virus sequences in pig faeces, though, with identities to circo- and cycloviruses. Although the detection of the novel virus indicates the suitability of the protocol for detection of so far unknown viruses, it has to be clearly stated, that only those viruses can be identified, for which sequence data of related viruses are present in the databases. For the identification of members of totally new virus families, novel analytical tools have to be developed using “deep bioinformatics”, e.g. functional predictions of putative proteins.

In summary, the results show that the established protocol enables the efficient analysis of the virus content in pig faeces including the identification of new viruses. The high percentage of bacteriophages detected in the faecal sample suggests an important function of these viruses in the modulation of the bacterial intestinal community, which should be analyzed in more detail in future. In addition, the clinical significance of the detected pig viruses - separate and in combination with each other – should be investigated further. The method may generally be useful in studies investigating aetiology, epidemiology and ecology of diseases. The implemented process control serves as quality control ensuring comparability of the method and may be used for further method optimization in future.

## Materials and Methods

### Ethics statement

The animal study was approved by the local ethic commitee of the state Berlin (Landesamt für Gesundheit und Soziales, Berlin, Germany) under the accession number G 0350/09.

### Faecal samples

Faecal samples were derived from pigs kept in the experimental animal facility of the Federal Institute for Risk Assessment (Berlin, Germany) and used in preliminary studies for establishment and optimization of the methods. For final testing of the established protocol, a fresh pooled faecal sample (approximately 100 g) was derived from five 35 day-old male pigs housed in one pen of the experimental facility. The animals were in good overall clinical condition. However, two of the five pigs suffered from watery diarrhoea at the time of the collection of the faeces. After collection, the faeces were stored at 4°C for 2 days until analysis. Individual rectal faecal samples and serum samples were collected at different time-points and stored at −80°C.

### Bacteriophages

The bacteriophages T4 (German Collection of Microorganisms and Cell Cultures, Braunschweig, Germany), M13 (kindly provided by E. Lanka, Max Planck Institute for Molecular Genetics, Berlin, Germany) and MS2 (kindly provided by J. Dreier, Ruhr University of Bochum, Bad Oeynhausen, Germany) were used as process control to assess the efficiency of the method. Table 1 summarizes the properties of the phages. M13 and T4 were propagated on E. coli JM110 (New England BioLabs GmbH, Frankfurt, Germany), MS2 on E. coli strain Top10 F+ (Invitrogen, Leek, Netherlands). For quantification, the phage titer of lysates was determined as previously described [42]. For the detection of the bacteriophage genomes at distinct steps of purification and amplification, quantitative real-time RT-PCR (qRT-PCR) assays were established. Primers and probes used for the detection of the phages are listed in Table 2. All qRT-PCRs were performed using the QIAamp probe RT-PCR kit (Qiagen, Hilden, Germany) in an ABI 7500 cycler (Applied Biosystems; Darmstadt, Germany). After reverse transcription at 50°C for 30 min and activation of the polymerase at 95°C for 15 min, 45 cycles were performed, each consisting of 94°C for 15 sec, 56°C for 1 min and 72°C for 1 min. Although the bacteriophages T4 and M13 have DNA genomes, their detection was also carried out using this RT-PCR protocol in order to enable parallel analysis of all three bacteriophage genomes using the same cycling conditions. Ten-fold diluted nucleic acid preparations of the titrated phages were used as standards for quantification.

### Purification and concentration of virus particles

Virus particles were purified and concentrated by a combination of tangential flow filtration (TFF), ultrafiltration and caesium chloride (CsCl) density gradient ultracentrifugation [17], [21], [31], [32], [43]. Briefly, approximately 100 g of pig faeces were resuspended in 1,000 ml SM-buffer by magnetic stirring. Thereafter, 1×10^7^ plaque-forming units of each bacteriophage were added to the suspension. Aliquots of the faecal suspension were taken before and after adding of phages and tested at a later date by qRT-PCR. The sample was centrifuged at 17,000× g for 30 min to remove the large particulate debris and the supernatant was collected. The procedure was repeated by a three hour centrifugation to remove smaller particular structures. Afterwards, a first TFF was performed using a 0.22 µm filter (PALL Corporation, Middleton; MA, USA) to remove bacterial and eukaryotic cells and debris. The remaining filtrate was subjected to a second TFF with a 50 kDa filter (PALL Corporation, Middleton; MA, USA) to concentrate the virus particles. This viral preparation was further concentrated by centrifugation through Vivaspin 50,000 MWCO concentrators (Sartorius Stedim Biotech GmbH, Goetting, Germany) at 1,500× g resulting in a final volume of 36 ml. Volumes of 18 ml each were loaded onto a stepwise CsCl density gradient with density layers of 1.7, 1.5, 1.35 and 1.2 g ml^−1^ (each 5 ml) and centrifuged at 65,000× g for 14 hours at 10°C. The 1.35–1.5 g ml^-1^ layers were collected from the gradients using a syringe and pooled resulting in a final volume of 9 ml.

### Nucleic acid preparation and deep sequencing

To eliminate free DNA present in the virus concentrate, 500 µl CsCl purified virus suspension were treated with 25 units DNase I (2,000 U/mg, bovine pancreas grad II; Roche Diagnostics GmbH, Mannheim, Germany) for 45 min at 37°C, followed by heat inactivation for 10 min at 65°C. Thereafter, DNA and RNA were simultaneously extracted from 200 µl using the RTP^®^ Pathogen Kit (Invitek; Berlin, Germany). The extracted nucleic acids (75 ng per reaction) were randomly primed for cDNA synthesis using the TransPlex^®^ Complete Whole Transcriptom Amplification Kit (WTA2, Sigma-Aldrich, St. Louis, MO, USA) according to the protocol recommended by the supplier; however, the annealing temperature was decreased to 40°C (cycles 1 and 2) and 45°C (cycles 3 and 4) to enable the simultaneous amplification of DNA and RNA. Aliquots of 75 µl each were removed from the WTA reaction at different cycle numbers, and purified as well as size-selected using MobiSpin S-400 Columns (MoBiTec, Goetting, Germany). The DNA concentration of the preparations was measured using a nanodrop spectrometer (PEQLAB Biotechnologie GMBH; Erlangen Germany) and the preparation derived from a minimum of amplification cycles with a DNA concentration higher than 50 ng/µl was used for deep sequencing. A total of 1 µg DNA was applied to deep sequencing on a 1/8 plate of the GS-FLX sequencer 454 Titanium (GS Titanium SV emPCR Kit (Lib-L) v2; GS Titanium PicoTiterPlate Kit 70×75; GS Titanium Sequencing Kit XLR70t; Life Sciences, Roche, Branford, USA) according to the manufacturer's protocol.

### Data analysis

Primary sequence analysis was applied to raw sequence reads, which were subjected to amplification primer/adaptor trimming using SeqMan (DNASTAR, Lasergene, Madison, USA) and selection for a minimum length of 50 nt. In parallel, all primary reads were subjected to *de novo* contig assembly using the 454 Newbler Assembler [44] software (http://www.my454.com/), with criteria of 90% minimum overlap identity and a minimum overlap length of 40 nt. Homology searches for primary reads and assembled contigs were performed with tBLASTx [45] and CLC Main Workbench 6.2 (http://www.clcbio.com/index.php?id=532) against a local database. This local database was created using EXCEL (2003). It included the viral genome non-redundant reference sequence nucleotide database (RefSeq, NCBI, ftp://ftp.ncbi.nih.gov/refseq/release/viral/ 30.08.2011 download) and additional sequences from recently discovered viruses, which had been manually added. BLAST results with an E-value < = 10^−4^ were selected and used for further grouping analysis, which was performed using a script manually written with R 2.13 [46]. This analysis included counting of detected species and determination of their taxonomy, which was also used to determine the virus hosts. Correction of the different genome length of the virus families was done by using the formula: formal size factor = read number/ genome size (the genome size for the respective virus species was derived from the above mentioned local database, which included these data from the RefSeq database, NCBI). The Shannon index [47] was calculated to compare the diversity of the species identified by primary reads and by assembled contigs.

### Whole genome analysis of PigSCV

Two overlapping fragments covering the whole genome sequence of PigSCV were amplified by PCR using nucleic acid derived from the concentrated virus particles as template. The Long range PCR kit (Qiagen, Hilden, Germany) was used with primers PigSCV-1s (5′-CCA ATC AGA TTC ACG CTT ACC G-3′) and PigSCV-1as (5 ′AAC ATC GTC AAC CGT ATC ATG G-3′) or primers PigSCV-2s (5′-GGG CCA CGC ATG AAC CTT CC-3′) and PigSCV-2as (5′-ACC ATT GAA ATC ATC TGG GAT G-3′) for amplification of overlapping fragments with lengths of 1,849 bp or 1,123 bp, respectively. The PCR products were directly sequenced in an ABI 3730 DNA Analyser (Applied Biosystems). The whole genome sequence was assembled from the sequenced fragments, submitted to the GenBank database under the accession number JQ023166. ORFs were predicted using the SeqBuilder module of the DNASTAR software package (Lasergene, Madison, USA). Sequence distances were calculated and phylogenetic trees were constructed with the MegAlign module implemented in the DNASTAR software package.

### Detection of PigSCV genome in pig samples

Faecal samples derived from the rectum of the pigs at d29, d33, d35 and d42 and serum samples of d42 were stored at −80°C. The nucleic acid was extracted from 200 µl faecal suspension or 100 µl serum using NucliSens Magnetic Extraction in a NucliSens EasyMag device (bioMerieux Deutschland GmbH, Nürtingen, Germany). PigSCV was detected by PCR using the TaKaRa ExTaq PCR Kit (TaKaRa Bio Europe, Göttingen, Germany) with primers PigSCV-2s and PigSCV-1as amplifying a 361 bp fragment of the PigSCV genome. PCR was performed by an initial denaturation step at 95°C for 5 min followed by 40 cycles each including 95°C for 30 sec, 60°C for 30 sec and 72°C for 45 sec, and a final elongation at 72°C for 5 min. PCR products were analysed by electrophoresis on ethidium bromide-stained agarose gels.

## References

[1] BMELV, Bundesministerium für Ernährung, Landwirtschaft und Verbraucherschutz; Federal Ministry of Food, Agriculture and Consumer Protection. Available: http://berichte.bmelv-statistik.de/SJT-8031700-0000.pdf

[2] Sencer DJ (2011). Perspective: Swine-origin influenza: 1976 and 2009.. Clin Infect Dis.

[3] Midgley SE, Bányai K, Buesa J, Halaihel N, Hjulsager CK (2011). Diversity and zoonotic potential of rotaviruses in swine and cattle across Europe..

[4] van der Poel WH, Verschoor F, van der Heide R, Herrera MI, Vivo A (2001). Hepatitis E virus sequences in swine related to sequences in humans, The Netherlands.. Emerg Infect Dis.

[5] Smith HW, Huggins MB (1983). Effectiveness of phages in treating experimental Escherichia coli diarrhoea in calves, piglets and lambs..

[6] Maidana-Giret MT, Silva TM, Sauer MM (2009). GB virus type C infection modulates T-cell activation independently of HIV-1 viral load.. AIDS.

[7] Roossinck MJ, Saha P, Wiley GB, Quan J, White JD (2010). Ecogenomics: using massively parallel pyrosequencing to understand virus ecology.. Mol Ecol.

[8] Margulies M, Egholm M, Altman WE, Attiya S, Bader JS (2005). Genome Sequencing in Open Microfabricated High Density Picoliter Reactors..

[9] Riesenfeld CS, Schloss PD, Handelsman J (2004). Metagenomics: Genomic Analysis of Microbial Communities.. Annu Rev Genet.

[10] Lamendella R, Domingo JWS, Ghosh S, Martinson J, Oerther DB (2011). Comparative fecal metagenomics unveils unique functional capacity of the swine gut.. BMC Microbiol.

[11] Arumugan M, Raes J, Pelletier E, Paslier DL, Yamada T (2011). Enterotypes of the human gut microbiome..

[12] Tang P, Chiu C (2010). Metagenomics for the discovery of novel human viruses.. Future Microbiol.

[13] Djikeng A, Kuzmickas R, Anderson NG, Spiro DJ (2009). Metagenomic analysis of RNA viruses in a fresh water lake..

[14] Li L, Pesavento PA, Shan T, Leutenegger CM, Wang C (2011). Viruses in diarrhetic dogs include novel kobuviruses and sapoviruses.. J Gen Virol 92 (Pt.

[15] Jere KC, Mlera L, O'Neill HG, Potgieter AC, Page NA (2011). Whole genome analyses of African G2, G8, G9, and G12 rotavirus strains using sequence-independent amplification and 454 pyrosequencing..

[16] Finkbeiner SR, Allred AF, Tarr PI, Klein EJ, Kirkwood CD (2008). Metagenomic analysis of human diarrhea: viral detection and discovery..

[17] Breitbart M, Hewson I, Felts B, Mahaffy JM, Nulton (2003). Metagenomic analyses of an uncultured viral community from human feces..

[18] Minot S, Sinha R, Chen J, Li H, Keilbaugh SA (2011). The human gut virome: Inter-individual variation and dynamic response to diet.. Genome Res.

[19] Reyes A, Haynes M, Hanson N, Angly FE, Heath AC (2010). Viruses in the faecal microbiota of monozygotic twins and their mothers..

[20] Victoria JG, Kapoor, Li L, Blinkowa O, Slikas B (2009). Metagenomic Analyses of Viruses in Stool Samples from Children with Acute Flaccid Paralysis..

[21] Zhang T, Breitbart M, Lee WH, Run JQ, Wei CL (2006). RNA Viral Community in Human Feces: Prevalence of Plant Pathogenic Viruses.. PLoS Biol 4 (1):.

[22] Shan T, Li L, Simmonds P, Wang C, Moeser A (2011). The fecal virome of pigs on a high-density farm..

[23] Palmenberg AC, Sgro JY (2005). Virus Particle Structures.. Fauquet CM, Mayo MA, Maniloff J, Desselberger U, Ball LA (eds) Virus Taxonomy: Eight Report of the International Committee on Taxonomy of Viruses.

[24] Cann AJ, Fandrich SE Heaphy S (2005). Analysis of the Virus Population Present in Equine Faeces Indicates the Presence of Hundreds of Uncharacterized Virus Genomes..

[25] Day JM, Ballard LL, Duke MV, Scheffler BE Zsak L (2010). Metagenomic analysis of the turkey gut RNA virus community.. Virol J.

[26] Li L, Shan T, Wang C, Côté C, Kolman J (2011). The Fecal Viral Flora of California Sea Lions..

[27] Phan TG, Kapusinszky B, Wang C, Rose RK, Lipton HL (2011). The Fecal Viral Flora of Wild Rodents..

[28] Dreier J, Störmer M Kleesiek K (2005). Use of Bacteriophage MS2 as an Internal Control in Viral Reverse Transcription-PCR Assays..

[29] Costafreda MI, Bosch A, Pintó RM (2006). Development, evaluation, and standardization of a real-time TaqMan reverse transcription-PCR assay for quantification of hepatitis A virus in clinical and shellfish samples..

[30] Ward P, Poitras E, Leblanc D, Letellier A, Brassard J (2009). Comparative analysis of different TaqMan real-time RT-PCR assays for the detection of swine Hepatitis E virus and integration of Feline calicivirus as internal control..

[31] Breitbart M, Salamon P, Andresen B, Mahaffy JM, Segall AM (2002). Genomic analysis of uncultured marine viral communities..

[32] Thurber RV, Haynes M, Breitbart M, Wegley L, Rohwer F (2009). Laboratory procedures to generate viral metagenomes..

[33] Johne R, Müller H, Rector A, van Ranst M, Stevens H (2009). Rolling-circle amplification of viral DNA genomes using phi29 polymerase..

[34] Kim Y, Chang KO, Straw B, Saif LJ (1999). Characterization of group C rotaviruses associated with diarrhea outbreaks in feeder pigs..

[35] Halaihel N, Masía RM, Fernández-Jiménez M, Ribes JM, Montava R (2010). Enteric calicivirus and rotavirus infections in domestic pigs..

[36] Pignatelli M, Moya A (2011). Evaluating the fidelity of de novo short read metagenomic assembly using simulated data..

[37] Lin Y, Li J, Shen H, Zhang L, Papasian CJ, Deng HW (2011). Comparative studies of de novo assembly tools for next-generation sequencing technologies..

[38] Blinkova O, Victoria J, Li Y, Keele BF, Sanz C (2010). Novel circular DNA viruses in stool samples of wild-living chimpanzees.. J Gen Virol 91 (Pt.

[39] Kim HK, Park SJ, Nguyen VG, Song DS, Moon HJ (2011). Identification of a novel single stranded circular DNA virus from bovine stool..

[40] Li L, Kapoor A, Slikas B, Bamidele OS, Wang C (2010). Multiple diverse circoviruses infect farm animals and are commonly found in human and chimpanzee feces..

[41] Ge X, Li J, Peng C, Wu L, Yang X (2011). Genetic diversity of novel circular ssDNA viruses in bats in China.. J Gen Virol 92 (Pt.

[42] Sambrook J, Russel D (2001). Molecular cloning: a laboratory manual..

[43] Breitbart M, Fekts B, Kelley S, Mahaffy JM, Nulton J (2004). Diversity and population structure of a near-shore marine-sediment viral community..

[44] Miller JR, Koren S, Sutton G (2010). Assembly algorithms for next-generation sequencing data..

[45] Altschul SF, Madden TL, Schäffer AA, Zhang J, Zhang Z (1997). Gapped BLAST and PSI-BLAST: a new generation of protein database search programs..

[46] R Development Core Team (2011). http://www.R-project.org/.

[47] Shannon CE (1997). A mathematical theory of communication..

